# Single-Cell Proteomic Profiling Identifies Nanoparticle Enhanced Therapy for Triple Negative Breast Cancer Stem Cells

**DOI:** 10.3390/cells10112842

**Published:** 2021-10-22

**Authors:** Wenzheng Wang, Bo Lei, Lin Li, Jianyu Liu, Zhihui Li, Yuheng Pang, Tong Liu, Zhigao Li

**Affiliations:** Harbin Medical University Cancer Hospital, Cancer Research Institute, Harbin Medical University, Harbin 150081, China; wangwenzheng@hrbmu.edu.cn (W.W.); 2359@hrbmu.edu.cn (B.L.); 202001398@hrbmu.edu.cn (L.L.); jyls865184@hrbmu.edu.cn (J.L.); lizhihui@hrbmu.edu.cn (Z.L.); pangyuheng@hrbmu.edu.cn (Y.P.)

**Keywords:** chitosan nanoparticle, spheroid cells, integrins

## Abstract

Breast cancer remains a major cause of cancer-related deaths in women worldwide. Chemotherapy-promoted stemness and enhanced stem cell plasticity in breast cancer is a cause for great concern. The discovery of drugs targeting BCSCs was suggested to be an important advancement in the establishment of therapy that improves the efficacy of chemotherapy. In this work, by using single-cell mass cytometry, we observed that stemness in spheroid-forming cells derived from MDA-MB-231 cells was significantly increased after doxorubicin administration and up-regulated integrin αvβ3 expression was also observed. An RGD-included nanoparticle (CS-V) was designed, and it was found that it could promote doxorubicin’s efficacy against MDA-MB-231 spheroid cells. The above observations suggested that the combination of RGD-included nanoparticles (CS-V) with the chemo-drug doxorubicin could be developed as a potential therapy for breast cancer.

## 1. Introduction

To date, breast cancer remains one of the most common cancer types and is a major cause of mortality worldwide [1]. Despite the advancements in diagnosis and the development of novel clinical treatments, chemotherapy has remained the mainstay of treatment for breast cancer patients [2,3,4]. However, many patients experience drug resistance and tumor relapse, which is a major challenge in current breast cancer chemotherapy [5].

Emerging evidence suggested that tumors are composed of heterogeneous populations, and a minute population of cancer cells known as cancer stem cells (CSCs) had been believed to contribute to drug resistance and cancer recurrence [6]. In mammary cancer, the aforementioned small population of cancer stem cells (BCSCs) aroused increasing attention, and BCSCs were reported to possess higher tolerability to chemotherapy [7,8,9]. The discovery of drugs targeting BCSCs was suggested to be an important advancement in the effort to establish therapy that improves the efficacy of chemotherapy [10,11].

Integrins are transmembrane proteins that act as cell surface receptors, mediating cell to cell and cell to ECM adhesion. It has been widely supported by many clinical studies that in most cells in the body, integrin αvβ3 levels are typically low but are highly expressed in breast cancer lesions and the endothelial cell surface [12]. Prospective identification studies have indicated that additional integrin αvβ3 is highly expressed in cancer stem cells [13]. Integrin αvβ3 was also reported to be involved in the resistance of carcinoma stem cells to EGFR inhibitors by promoting stemness [14]. These observations potentially implicate the αvβ3 integrin in the positive regulation of stemness and suggest that it could be developed as an anti-BCSCs target.

Integrin αvβ3 strongly binds to the arginine-glycine-aspartic acid (RGD) tripeptide sequence; therefore, RGD peptides can be used as drug or drug delivery tools in treating integrin αvβ3 expressing tumors and tumor vasculature cells [15,16]. Combining RGD peptides with nanotechnology could preferentially inhibit tumor tissues in a targeted manner without affecting normal tissues. Due to the development of nanotechnology, numerous RGD-included peptide nanoparticles have been widely explored in regard to cancer treatment. Here, we developed an RGD-included peptide chitosan nanoparticle, which we hypothesized could augment chemotherapy efficacy against triple-negative breast cancer stem cells derived from MDA-MB-231 cells.

## 2. Materials and Methods

### 2.1. Reagents and Chemical

Doxorubicin (purity > 98%) was purchased from Sigma-Aldrich (St. Louis, MO, USA). An annexin V-FITC staining Apoptosis Detection Kit (BD Biosciences Pharmingen, San Diego, CA, USA) was used to detect apoptotic cells. Primary antibodies were used as follows: PE-CD133 (S16015F, Biolegend, San Diego, CA, USA), FITC-CD90 (MRC OX-7, Abcam, Cambridge, UK), FITC-integrin α_v_β_3_ (23C6, Biolegend). Antibodies conjugated with metal that were used in CyTOF analysis are listed in Table 1.

### 2.2. Preparation and Characterization of Chitosan-Modified Peptide Nanoparticles

Chitosan (MW < 3000; degree of deacetylation of 90%) was purchased from Golden-Shell Biochemical Co., Ltd. CRGDV quintapeptide was synthesized by GL Biochem (Shanghai) Ltd. N-succinimidyl-3-(2-pyridyldithio)-propionate (SPDP), and Tris (2-chloroethyl) phosphate were purchased from Sigma-Aldrich. To prepare the chitosan-modified RGDV peptide, N-succinimidyl-3-(2-pyridyldithio)-propionate (SPDP) was used as a difunctional linking agent, using the method described in a previous publication [17]. Briefly, SPDP was dissolved in DMSO to the concentration of 20 nm, then reacted with CS aqueous solution for 1 h with the molar ratio of 3:1. Meanwhile, CRGDV peptide was dissolved into PBS, co-incubated with TCEP to achieve the final concentration of 4 mm. After removing residual non-reacted SPDP using a desalting column and washing byproducts using PBS, the mixture was mixed with the peptide at the molar ratio of 1:3 and was next incubated with stirring overnight and washed 4 times using a desalting column for purification with distilled water.

The size and zeta potential of CS-Vs were analyzed by the dynamic light scattering (DLS) technique using the Zetasizer (Malvern Instruments, Malvern, Germany) at a fixed scattering angle of 90° and a temperature of 25 °C. The IR spectra of the CS, CRGDV, and CS-V were obtained using KBr pellets on an FTIR spectrophotometer in the range between 200 and 4000 cm^−1^. Transmission electron microscopy (TEM) and scanning electron microscopy (SEM) were used to observe the morphology of prepared nanoparticles.

### 2.3. MDA-MB-231/4T1 Cells and Spheroid Cells Culture

Human triple-negative breast cancer cell line MDA-MB-231 and mouse triple-negative breast cancer cell line 4T1 were purchased from ATCC and cultured in DMEM medium with 10% fetal bovine serum and was aired with 5% CO_2_ at 37 °C. Single MDA-MB-231 cells or 4T1 cells were plated in low attachment 6-well plates (Corning, Oneonta, NY, USA) at a density of 5000 cells/well, and cultured in serum-free DMEM/F12k containing B27 (Thermo Fisher Scientific, Inc., Waltham, MA, USA), epidermal growth factor (EGF, 20 ng/mL; Solarbio Science and Technology Co., Ltd., Beijing, China), and basic fibroblast growth factor (bFGF20 ng/mL, Solarbio Science and Technology Co., Ltd., Beijing, China). The cells were allowed to grow for 7 days; then, the medium was changed, and they were cultured for another 7 days. The quantification of the number of spheres was carried out using microscopy to count all spheres with a diameter bigger than 50 μm. Experiments were repeated three times, with three replicates each.

### 2.4. CCK8 Assay Detected Drug Sensitivity

Cell viability was evaluated by using a CCK8 assay. In brief, single cells were seeded in 96-well plates (4 × 10^3^ cells/well) and cultured overnight before being treated with a different concentration of doxorubicin (0.1625, 0.325, 0.625, 1.25, 2.5, and 5 μm), and the same concentration of doxorubicin combined with 20 ng/mL CS-V, for 48 h. After adding 10 μL of CCK8 solution, the plates were incubated at 37 °C for 4 h before the absorbance was measured at 490 nm using a microplate reader (Bio-Rad Laboratories, Hercules, CA, USA).

### 2.5. Immunofluorescence Staining

Immunofluorescence (IF) for CD133, CD90, and integrin α_v_β_3_ was performed on breast cancer cells and/or cultured spheroids using fluorescence-conjugated primary antibodies. In brief, cells were harvested after treatment and fixed in 1.6% paraformaldehyde for 10 min, R.T, and blocked with 1% normal goat serum and incubated with the above antibodies for 30 min at room temperature in the dark before being detected using BD Calibur flow cytometry. A total of 10,000 events per sample were acquired, and fluorescence data were recorded.

### 2.6. Time-of-Flight Mass Cytometry

Cultured breast cancer cells and spheroid cells were harvested and washed twice with ice-cold PBS after being treated with medium, doxorubicin, CS-V, and CS-V plus doxorubicin for 48 h. Single cells were then incubated with 1 mL 2 µM of cisplatin (Fluidigm, San Francisco, CA, USA) for 2 min to label death cells. FIX I (Fluidigm, San Francisco, CA, USA) solution was used to fix the cells for 15 min at R.T. After being washed three times with CSB (Cell Staining Buffer, Fluidigm, San Francisco, CA, USA), cells were stained with an antibodies cocktail against surface markers (Table 1) for 0.5 hr at room temperature, followed by being washed three times with CSB. Cells were then permeabilized with 80% methanol at 4 °C for 15 min and washed three times with CSB; then, all cells were subsequently stained with an antibodies cocktail against intracellular markers. After being washed three times with CSB, cells were stained with iridium-containing DNA intercalator (^191^Ir/^193^Ir, final concentration of 125 nM) in FIX and Perm (Fluidigm, San Francisco, CA, USA) solution for 1 hr at room temperature. Cells were then washed three times with double-distilled water and resuspended in 10% EQ Four Element Calibration beads (Fluidigm, San Francisco, CA, USA) solution before being detected using a Helios mass cytometer (Fluidigm, San Francisco, CA, USA). All the normalized .fcs (Flow Cytometry Standard) files were then uploaded to Cytobank (https://www.cytobank.org/ accessed on 25 July 2021) to finish data cleaning and SPADE analysis. Based on normalized expression levels of the included protein markers (Table 1), CyTOF data were clustered and visualized using the t-distributed stochastic neighbor embedding (t-SNE) algorithm.

### 2.7. Statistical Analysis

The bioassay results were expressed as means ± standard deviation (SD). At least three samples were prepared for assays of every attribute. Data analysis was performed using an ANOVA test. P-values less than 0.05 were considered to be statistically significant.

## 3. Results

### 3.1. Prepared and Characterization of Chitosan Nanosized Peptide

**Figure 1:**
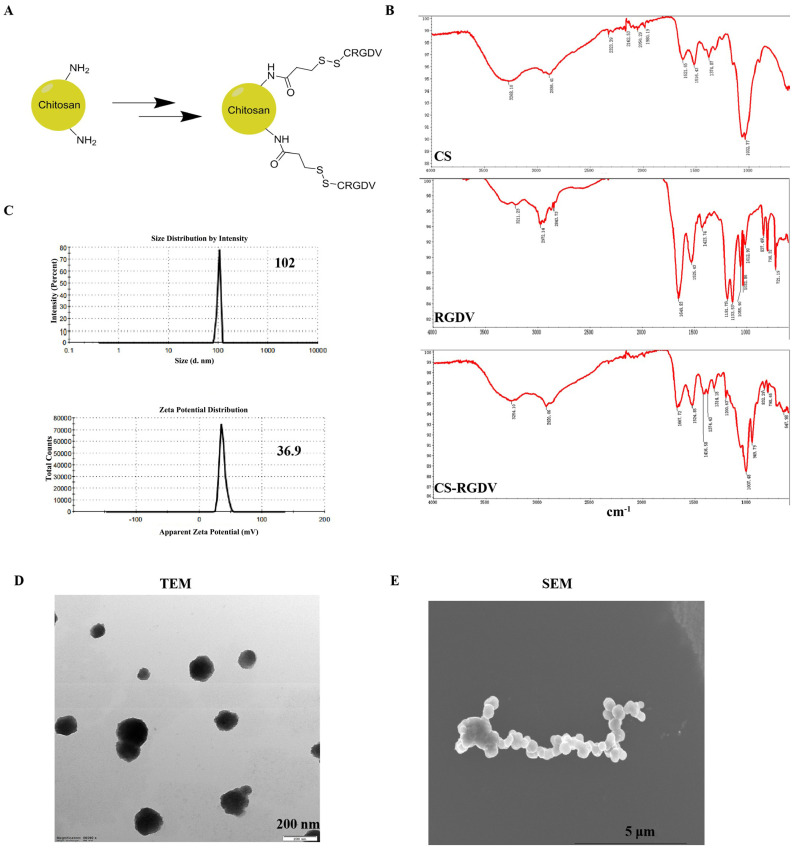
Characterization of chitosan-modified peptide nanoparticle. (**A**) Schematic illustration of the synthesis process of chitosan-modified peptide nanoparticles. (**B**) IR spectrum of RGDV peptide, chitosan (CS), and chitosan-modified peptide (CS-V). (**C**) Size distribution and zeta potential of CS-V. (**D**) TEM image of CS-V. (**E**) SEM image of CS-V.

A chitosan-modified nanosized peptide was designed and synthesized. Chitosan is a promising nano-carrier in anticancer research, which was confirmed by numerous studies. In this work, we used N-succinimidyl-3-(2-pyridyldithio)-propionate (SPDP) as a linker and conjugated CRGDV five peptides on chitosan and formed nanoparticles (Figure 1A). To study the chemical structure properties of chitosan and chitosan-modified peptide nanoparticles, Fourier transform infrared (FTIR) analyses were performed (Figure 1B). As the FTIR spectrum shows, for CS itself, peaks observed at 1032 cm^−1^ represent the stretching of C-O groups, and a broad band in 3268 cm^−1^ arises from the overlap of the OH and NH2 groups in CS [18]. The main characteristic band for amide groups of the CRGDV peptide could be observed at 1649 cm^−1^. In the spectrum of CS-V, considerable changes in the shape and frequencies of the bands imply that the peptide was effectively linked with chitosan. The average size of CS-V nanoparticles (0.1 mg/mL) was about 102 nm (PDI was 0.437, Figure 1C) with positive zeta potential (36.9 mV) (Figure 1C). Transmission electron microscopy (TEM) images of CS-V nanoparticles (Figure 1D) revealed a spherical morphology, with average sizes of about 180 nm. The morphology of the CS-V nanoparticle was also explored by using SEM, as shown in Figure 1E.

### 3.2. MDA-MB-231-Cultured Spheroid Cells Exhibit BCSC-like Properties, and Administration of Doxorubicin Could Enhance Stemness in Spheroid Cells

**Figure 2:**
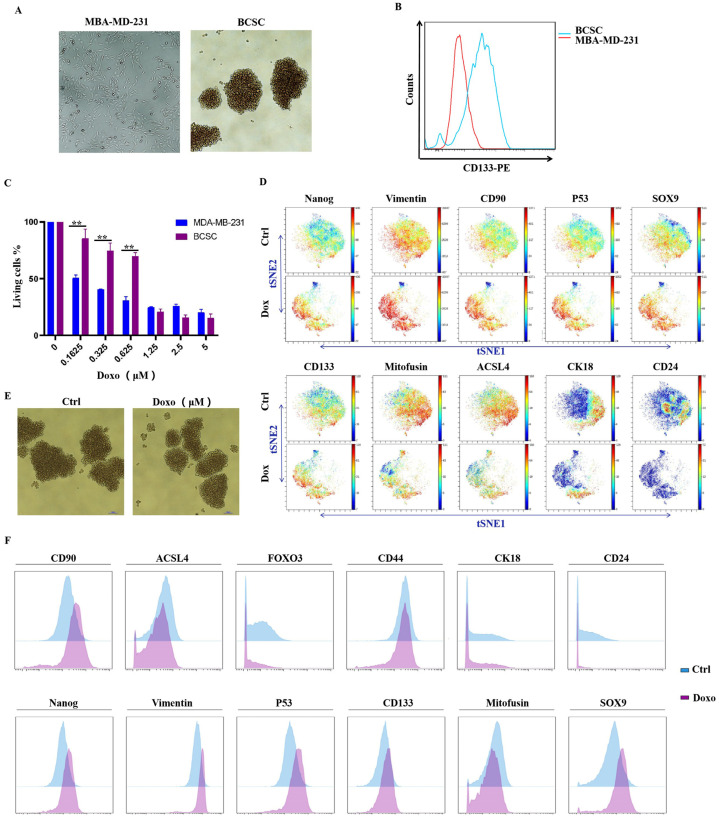
MDA-MB-231-derived spheroid cells were resistant to doxorubicin administration, and stem cell markers were dramatically induced. (**A**) Images of MDA-MB-231 cells and cultured spheroids. (**B**) Flow cytometry detected stemness marker CD133 expression of MDA-MB-231 cells and BCSCs. (**C**) CCK8 assay analysis showed anti-proliferation of doxorubicin against MDA-MB-231 cells and MDA-MB-231-derived spheroid cells. (**D**) t-SNE plot cluster distribution, and protein markers including CD133, CD90, vimentin, Nanog, Sox9, CK18, and CD24 expression of spheroid cells before and after being treated with doxorubicin (blue-to-red scale, red represents high expression). (**E**) Images of cultured stem cell spheres treated with or without doxorubicin. (**F**) Protein markers’ mean expression level of BCSCs treated with or without doxorubicin. (** *p* < 0.01, *n* = 3 biologically independent samples per group).

In accordance with previous publications, we prepared spheroid-forming cells derived from MDA-MB-231 breast cancer cells. Images of spheroids in culture were taken using light microscopy via the 4× objective. As shown in Figure 2A, bright field images of MDA-MB-231 cells grew with clear and sharp boundaries between them, whereas the spheroids cells grew into non-adherent spheres within 14 days.

To evaluate the stemness of cultured spheroids, CD133 protein expression, which is routinely used for the identification of stem cells, was examined by flow cytometry. The results are shown in Figure 2B; the CD133 expression of spheroid cells was significantly increased compared to the MDA-MB-231 parental cells. The results indicated that cultured spheroid cells presented typical stem cell properties. We then detected the anti-proliferation activity of doxorubicin to MDA-MB-231 parental and spheroid cells. As shown in Figure 2C, spheroid cells displayed strong resistance to doxorubicin compared to parental cells, as IC50 values increased from 0.12 ± 0.02 to 0.77 ± 0.10 μM. Figure 2E displays before and after images of spheroid cells treated with doxorubicin (0.8 μM).

To further study the impact of doxorubicin on stem-like properties of MDA-MB-231 spheroid cells, a single-cell time-of-flight mass cytometry (CyTOF) analysis was performed. CyTOF is an emerging technique that allows multiple cellular proteins to be simultaneously analyzed at the single-cell level. To identify a cancer stem-cell-like subpopulation in MDA-MB-231 spheroid cells, we included stem cell markers, such as CD133, CD90, CD44, and CD24, and stem cell transcription factors such as SOX9 and Nanog. Considering the fact that EMT (epithelial-mesenchymal transition) is often related to the acquisition of stemness characteristics [19], EMT markers such as E-cadherin and vimentin were also included. The results are displayed as a 2-D t-SNE plot map, as shown in Figure 2D. As we can see from the map, cells treated with doxorubicin were distributed in the opposite direction compared to the control group, which indicated that they had a significantly different expression pattern. In the doxorubicin group, the expression of stemness markers, such as CD90, SOX9, Nanog, and CD133, as well as the EMT marker vimentin, was dramatically up-regulated; however, Mitofusin, ACSL4, CD24, and CK18 protein levels were decreased. These results suggest that stem-like cells, as well as EMT, are highly induced by doxorubicin administration in heterogeneous populations. P53 protein was also found to be increased by doxorubicin. Even though P53 was commonly recognized as a tumor suppressor, recently, it was reported to act as a critical mediator of chemotherapy drug-induced cancer stem cell activation via the WNT/β-catenin signaling pathway [20]. The mean expression of those protein markers is also displayed using a histogram in Figure 2F.

### 3.3. Integrin αvβ3 and CD90 Expression Was Significantly Induced by Doxorubicin, and Integrin αvβ3-Targeted CS-V Nanopeptide Could Promote Doxorubicin Efficacy against MDA-MB-231 Spheroid Cells

**Figure 3:**
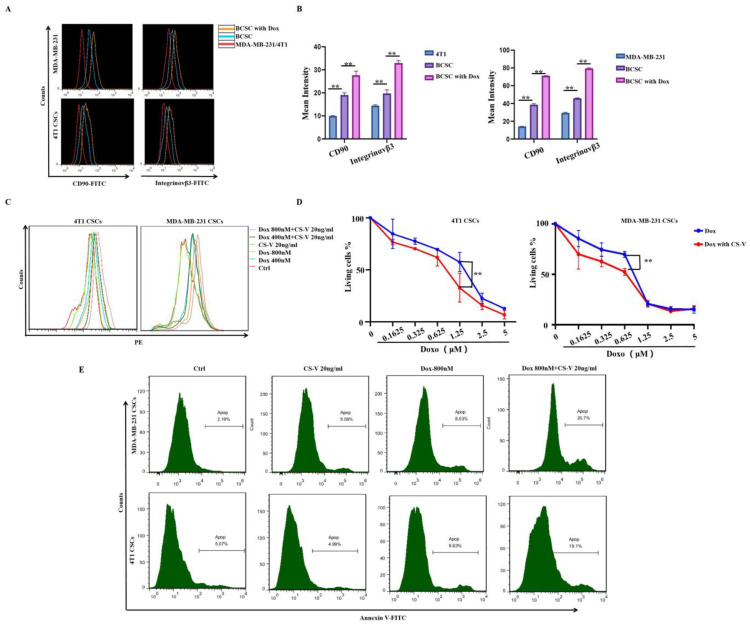
CS-V nanoparticles enhanced the activity of doxorubicin against BCSCs. (**A**) Flow cytometry results of the surface marker CD90 and integrin α_v_β_3_ expression induced by doxorubicin in BCSCs derived from MDA-MB-231 and 4T1 cells. (**B**) Statistical analysis of CD90 and integrin α_v_β_3_ expression induced by doxorubicin in BCSCs derived from MDA-MB-231 and 4T1 cells. (**C**) Flow cytometry analysis revealed that doxorubicin uptake was increased by CS-V nanoparticles. (**D**) Anti-proliferation activity of doxorubicin against BCSCs was enhanced by CS-V nanoparticles. (**E**) AnnexinV-FITC staining detected by flow cytometry revealed that apoptotic death induced by doxorubicin in BCSCs was enhanced by CS-V nanoparticles. (** *p* < 0.01, *n* = 3 biologically independent samples per group).

Among all the increased stemness markers, CD90 aroused our attention. CD90 was reported as a stem cell marker that had an association with a number of cell surface molecules that allow the formation of extra/intracellular multiprotein complexes that could promote cancer formation in various cancer cell lines [21,22,23]. CD90-α_V_β_3_ integrin interaction is important for cancer cell migration, invasion, and transvasation [24]. β3 integrin was also reported to be required for the promotion of sphere formation and up-regulated the expression of the cancer stem cell marker CD133 by CD90 [25]. Additionally, integrins, including the β_3_ subset, have been reported to be involved in mediating drug resistance in different cancers [26]. In our study, a fluorescence flow cytometry assay was performed to detect α_V_β_3_ integrin expression that was induced in MDA-MB-231- and 4T1-derived spheroid cells by doxorubicin. The results are shown in Figure 3A, B; CD90 and α_V_β_3_ integrin were up-regulated in both spheroid cells compared to MDA-MB-231, and 4T1 parental cells, which could be highly induced by doxorubicin. Thus, we hypothesized that α_V_β_3_ integrin up-regulation might be involved in the promoted cancer stemness and chemo-resistance induced by doxorubicin in MDA-MB-231 and 4T1 spheroid cells.

To address our hypothesis, we prepared a nanosized α_V_β_3_ integrin antagonist named CS-V, as described above, and evaluated its activity regarding the promotion of doxorubicin efficacy against MDA-MB-231 and 4T1 spheroid cells. As shown in Figure 3C, fluorescence intensity revealed that the intracellular uptake of doxorubicin was promoted by CS-V nanoparticles, with a final concentration of 20 ng/mL. By adding the same concentration of CS-V nanoparticles, the anti-proliferation activity of doxorubicin against MDA-MB-231 and 4T1 spheroid cells was significantly enhanced, as shown in Figure 3D. Annexin V-FITC staining revealed that apoptotic cell death induced by doxorubicin was significantly enhanced when in combination with CS-V nanoparticles.

Overall, our results indicated that MDA-MB-231- and 4T1-derived spheroid cells were resistant to doxorubicin, and α_V_β_3_ integrin targeting nanoparticles could enhance doxorubicin efficacy.

### 3.4. Visualizing CS-V-Promoted Doxorubicin against BCSCs at Single-Cell-Level

**Figure 4:**
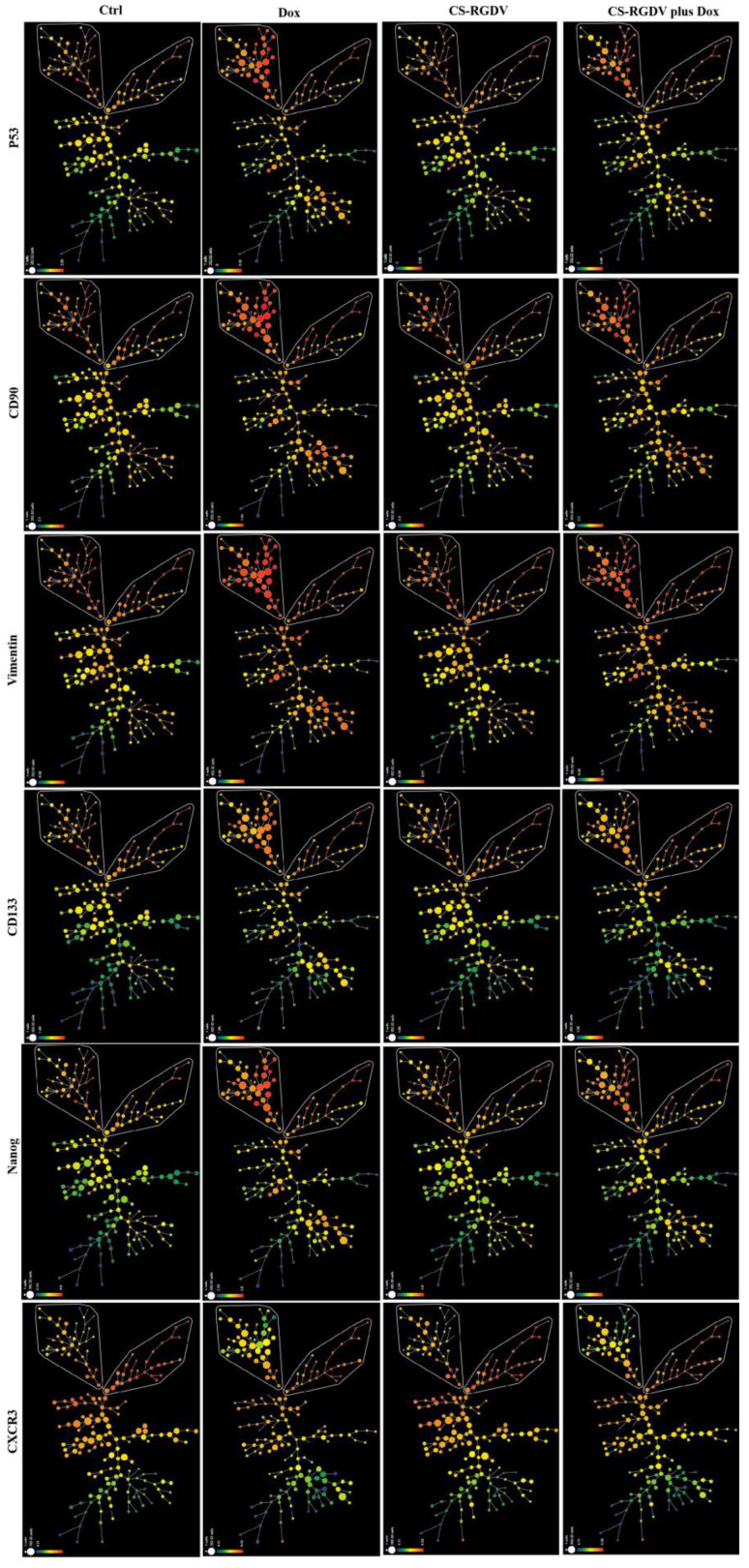
SPADE analysis (green-to-red scale, red represented high expression) showed stemness induced by doxorubicin was decreased in combination with CS-V nanoparticles. All the nodes in the figures represent a group of cells with similar protein expression patterns, and the size of the node represents the frequency of this group of cells.

**Figure 5:**
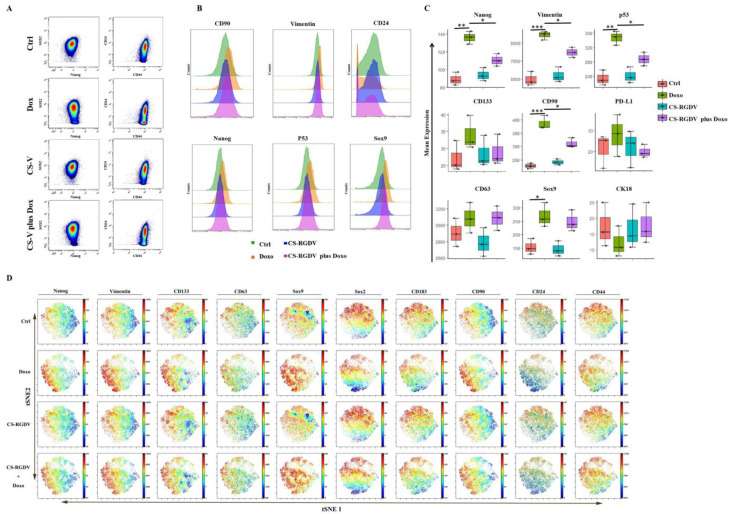
In-depth analysis of nanoparticles enhances the activity of doxorubicin. (**A**) Dot plots display cell subsets’ distribution of BCSCs in different treatment groups. (**B**) Histograms show the expression of CD90, vimentin, Nanog, P53, and Sox9 were up-regulated by doxorubicin and could be inhibited with a combination of CS-V nanoparticles. (**C**) Boxplots showing mean expression of protein markers expression in different groups. (**D**) t-SNE heatmap of markers’ expression in different treatment groups (blue-to-red scale, red represented high expression). (* *p* < 0.05, ** *p* < 0.01, *** *p* < 0.001, *n* = 3 biologically independent samples per group).

For the analysis of the phenotypic heterogeneity of MDA-MB-231 spheroid cells induced by doxorubicin and nanoparticles combined with doxorubicin, we performed spanning tree progression (SPADE) analysis, which is commonly used for high-dimensional cytometry data by profiling cells with common protein expression into the same subgroup. The colors in SPADE maps are used to visualize the average intensity of marker expression (red, high; green, low), and the size of the node in a SPADE map represents the frequency of this subgroup. According to our data that are partially listed in Figure 4, untreated MDA-MB-231-derived spheroid cells developed a certain number of stem-like cells with higher CD133, CD90, P53, Nanog, and vimentin expression, shown on the top of the map and circled as two different subpopulations. The difference between them lies in CXCR3 expression, a chemokine receptor that reportedly contributes to the metastasis of breast and other cancers [27]. We also found an interesting phenomenon that doxorubicin could only increase stem-like subpopulations with lower expressions of CXCR3 on the upper left corner and up-regulate CD133, CD90, vimentin, and p53 expression. In combination with CS-V nanoparticles, the up-regulated stemness protein markers and subpopulation could be suppressed. We also used dot plots to identify the distribution of cell subsets in different treatment groups; as shown in Figure 5A, doxorubicin-induced Nanog expression and increased the proportion of the CD44^+^CD24^−^ subpopulation that could be suppressed by adding CS-V nanoparticles. Histograms displaying the expression of CD90, vimentin, Nanog, P53, and Sox9 were up-regulated by doxorubicin and could be inhibited with the combination of CS-V nanoparticles (Figure 5B). The statistical results of marker expression are also displayed using boxplots in Figure 5C; doxorubicin-induced high expression levels of Nanog, vimentin, p53, CD133, CD90, which were effectively suppressed by CS-V nanoparticles. Data generated by CyTOF analysis were identified using single-cell proteomic analysis [28,29,30]. Thus, we performed advanced analysis using the t-distributed stochastic neighbor embedding (t-SNE) algorithm to visualize every single cell (blue-to-red scale, red represented high expression). As shown in Figure 5D, the t-SNE dot plot revealed heterogeneous populations within MDA-MB-231 spheroid cells with or without doxorubicin treatment.

## 4. Discussion

Breast cancer remains a major cause of cancer-related deaths in women worldwide. One of the most aggressive subtypes of breast cancer is triple-negative breast cancer (TNBC). Chemotherapy remains the mainstay of clinical treatment against TNBC; however, chemo-drug-promoted stemness and enhanced stem cell plasticity in TNBC are causes for great concern due to their contribution to chemo-resistance, cancer relapse, and increased aggression. Thus, developing therapeutic interventions against chemo-drug-induced stemness could potentially benefit triple-negative breast cancer patients.

Integrin-αvβ3-mediated stemness had been linked to the resistance of breast, lung, and pancreatic cancer to the EGFR inhibitor erlotinib in recent studies [31,32,33,34]. There is a consensus recognition that integrin-αvβ3 could be targeted to improve therapy against cancers. In our study, integrin αvβ3 expression was increased in spheroid-forming cells compared with triple-negative breast cancer cell MDA-MB-231 cells and highly up-regulated by doxorubicin, a commonly used chemo-drug in the clinical treatment of TNBC. Since studies reported that doxorubicin resistance in breast cancer mainly results from cancer stem cell-like cells [11], integrin-αvβ3 might be a potential target for the improvement of doxorubicin treatment.

According to our results, the cultured spheroid cells exhibit BCSC-like properties and are resistant to doxorubicin. Furthermore, based on our CyTOF data, the expression of stemness markers, such as CD90 and CD133, as well as the EMT marker vimentin, were dramatically up-regulated by doxorubicin in spheroid cells. On the other hand, CK18 and CD24 expression were decreased. Cytokeratin 18 (CK18) is a major intermediate protein that is essential for the maintenance of cellular structures; additionally, CK18 down-regulation has been considered to be a hallmark of EMT. In a previous publication, CD90 was reported to promote sphere formation in vitro and up-regulate the expression of the cancer stem cell marker CD133 via β3 integrin. Meanwhile, inactivation of the integrin αvβ3 pathway could reverse EMT and chemo-drug resistance in breast cancer cells [35]. Thus, integrin αvβ3 expression might play an important role in spheroid cells resistant to doxorubicin.

RGD peptides are targeting ligands that specifically bind to integrin αvβ3. In this work, we designed an RGD-included nanoparticle (CS-V) and discovered that this nanoparticle could augment doxorubicin efficacy against triple-negative breast cancer stem cells. According to our data from the CyTOF assay, the doxorubicin-induced expression of Nanog, vimentin, CD133, and CD90 was effectively suppressed when combined with CS-V nanoparticles. These observations indicated the possibility that targeting integrin αvβ3 by RGD peptide included nanoparticles can be developed as anticancer strategies to overcome chemo-drug-induced stemness and drug resistance.

In our study, the level of P53 protein was also found to be increased by doxorubicin in spheroid cells and could be inhibited by CS-V. Even though P53 is commonly recognized as a tumor suppressor, it was recently reported to act as a critical mediator of chemotherapy-drug-induced cancer stem cell activation via the WNT/β-catenin signaling pathway [20]. Therefore, the molecular mechanisms of P53 protein in the doxorubicin-induced stemness of breast cancer can be further explored.

In summary, the above findings have implications for the understanding of doxorubicin-induced stemness in triple-negative breast cancer and offer a new possible therapeutic strategy for targeting integrin αvβ3 in triple-negative breast cancer.

## Figures and Tables

**Table 1:** Antibodies used for CyTOF.

Antigen	Mass	Antibody Clone	Source
NANOG	142	23D2-3C6	Biolegend
VIMENTIN	143	AF2105	R&D
P53	144	AF1043	R&D
CD133	145	MAB11331	R&D
CD51/CD61	168	23C6	Biolegend
CD63	146	H5C6	Biolegend
SOX9	153	sc-166505	Santa Cruz
SOX2	155	MAB2018	R&D
CD183	156	MAB160	R&D
Mitofusin	149	sc-166644	Santa Cruz
CD90	159	5E10	Biolegend
PDL1	209	MAB1561	R&D
INTEGRIN b7	162	FIB27	Biolegend
ASMA	163	MAB1420	R&D
E-CADHERION	164	sc-8426	Santa Cruz
NFATC1	154	7A6	Biolegend
MUSASHI-1	155	3F8-E6-B9	Biolegend
CK18	167	sc-32329	Santa Cruz
CD24	169	ML5	Biolegend
FOXO3	170	sc-48348	Santa Cruz
CD44	171	C44Mab-5	Biolegend
53BP2	172	sc-398311	Santa Cruz
OCT4	146	3A2A20	Biolegend
β-catenin	147	AF1329	R&D
E2F1	148	AF4825	R&D

## Data Availability

The data that support the findings of this study are avilable from the corresponding atuthor upon reasonable request.
